# Ophthalmic Screening in Patients with Coronavirus Disease 2019: A Prospective Cohort Study

**DOI:** 10.3390/jcm10050896

**Published:** 2021-02-24

**Authors:** Anthia Papazoglou, Anna Conen, Sebastian Haubitz, Markus Tschopp, Viviane J. Guignard, Marcel N. Menke, Tim J. Enz

**Affiliations:** 1 Department of Ophthalmology, Cantonal Hospital Aarau, 5000 Aarau, Switzerland; markus.tschopp@ksa.ch (M.T.); viviane.guignard@ksa.ch (V.J.G.); marcel.menke@ksa.ch (M.N.M.); 2 Department of Infectious Diseases and Hospital Hygiene, Cantonal Hospital Aarau, 5000 Aarau, Switzerland; anna.conen@ksa.ch (A.C.); sebastian.haubitz@ksa.ch (S.H.); 3 Department of Internal and Emergency Medicine, Cantonal Hospital Aarau, 5000 Aarau, Switzerland; 4 Department of Ophthalmology, Inselspital, Bern University Hospital, University of Bern, 3010 Bern, Switzerland; 5 Department of Clinical Neuroscience, Section of Ophthalmology and Vision, St. Erik Eye Hospital, Karolinska Institutet, 171 64 Stockholm, Sweden

**Keywords:** SARS-CoV-2, COVID-19, ocular involvement, retina, optic nerve

## Abstract

Postmortem pathological examinations, animal studies, and anecdotal reports suggest that coronavirus disease 2019 (COVID-19) could potentially affect intraocular tissue. However, published evidence is scarce and conflicting. In our study, we screened 100 eyes of 50 patients hospitalized for COVID-19. Relevant medical and ophthalmological history was assessed as well as symptoms, laboratory results, specific treatments, clinical course, and outcome. Ophthalmic exams including assessment of best corrected visual acuity (BCVA), intraocular pressure (IOP), color perception, ocular motility, ophthalmoscopy as well as optical coherence tomography (OCT) of the macula and the optic disc was performed at hospital admission and 29 to 192 days later. Of the 50 patients included, 14 (28%) were female. Median age was 64.5 (range 29–90) years. COVID-19 severity was mild in 15 (30%), severe in 30 (60%), and critical in five cases (10%). At baseline, median BCVA was 0.1 (0–1.8) Logarithm of the Minimum Angle of Resolution (LogMAR) and median IOP was 16 (8–22) mmHg. At follow-up, no relevant changes in BCVA and IOP were documented. No signs of active intraocular inflammation or optic nerve affection were found and OCT findings were widely stable during the observation period. Our findings suggest that COVID-19 does not regularly affect intraocular tissue.

## 1. Introduction

In 2019, severe acute respiratory syndrome coronavirus 2 (SARS-CoV-2) was discovered and identified as the cause of a novel, emerging illness that came to be termed as coronavirus disease 2019 (COVID-19). After first isolated in China, SARS-CoV-2 spread pandemically across the globe within only a few weeks, infecting millions of people up to date [1]. COVID-19 was initially considered a condition characterized predominantly by respiratory symptoms. However, it became rapidly evident that COVID-19 is in fact a systemic illness with various potential extrapulmonary manifestations and complications [2]. From the early phases of the pandemic, studies have indicated that SARS-CoV-2 frequently causes ocular surface irritation and may also be transmitted via the conjunctival route [3]. Questions quickly arose whether other ocular tissues could also be affected [4]. Indeed, animal studies suggest that SARS-CoV-2 could potentially damage the retina and the optic nerve. In mice, some coronavirus strains have been shown to disrupt the blood-retinal barrier, inducing retinitis and optic neuritis with significant axonal loss over a few weeks [5,6]. Anosmia and dysgeusia are widely reported symptoms in COVID-19, indicating that the disease can affect sensory perception [2]. In addition, angiotensin converting enzyme-2 (ACE-2), the major binding protein for SARS-CoV-2, has been isolated on the ocular surface, in the uvea, and the retina [7,8]. Moreover, SARS-CoV-2 ribonucleic acid has been detected in ocular biopsies of deceased patients with COVID-19 [9]. The possibility of optic nerve and intraocular manifestation is therefore an obvious fear.

A number of anecdotal reports suggest possible neuro-ophthalmic involvement in COVID-19 including ocular motility dysfunction, diplopia, and ptosis [10]. Intraocular, especially posterior segment examination, require ophthalmological expertise and technical equipment that is usually not available in an infectious disease unit. Hence, evidence on intraocular involvement in COVID-19 is scarce, originating mainly from a few letters to the editor, case reports, and small case series [11]. Concerns have been expressed that major neuro-ophthalmological and intraocular manifestations are underreported as a result of limited diagnostic capabilities [10]. In addition, the sparse reports on intraocular findings in COVID-19 are conflicting and highly controversial [11]. Marinho et al. described retinal abnormalities assessed by optical coherence tomography (OCT) in 12 relatively young patients with COVID-19, predominantly not in need of hospital care [12]. Thereby, the authors left the impression that SARS-CoV-2 regularly affects intraocular segments, even in milder cases. The report provoked a number of reactions claiming that the findings may only represent correlates of physiologic vessels and retinal nerve fiber layer myelination [13,14,15]. Zapata and colleagues documented diminished retinal vessel densities in patients recovered from SARS-CoV-2 infection [16], while Invernizzi et al. measured amplified retinal vessel diameters on fundus photographs from patients with severe disease [17]. Other authors have described retinal hemorrhages and cotton wool spots in a minor, yet significant proportion of patients with COVID-19 [18,19]. Such retinal findings, however, are highly unspecific and therefore prone to be confounded by prevalent cardiovascular co-morbidities. Indeed, in another retrospective review of fundus photographs of COVID-19 patients, no evidence of retinal lesions was found [20]. Thus, the nature and frequency of intraocular involvement in COVID-19 is yet to be clarified [15], calling for a “thorough ophthalmological examination in a representative cohort of patients” [9].

The purpose of our study was to systematically, comprehensively, and longitudinally investigate possible ocular manifestations in a cohort of patients hospitalized for COVID-19 between hospital admission and one to three months later.

## 2. Materials and Methods

Consecutive patients hospitalized for COVID-19 at the Cantonal Hospital Aarau in Switzerland, a tertiary care referral center, were enrolled. SARS-CoV-2 infection was confirmed primarily by polymerase chain reaction (PCR) using a nasopharyngeal swab. In patients with a negative PCR result, but suggestive clinical and radiological presentation, serologic antibody testing was performed. In the case of a positive serologic result, diagnosis of COVID-19 was considered validated, as a history of previous infection was unlikely at this early stage of the pandemic. At the time of hospital admission, full medical and ophthalmological history was taken including previous systemic and ocular diseases as well as ophthalmic surgical interventions. Symptoms suggestive for COVID-19 (fever, respiratory symptoms, taste and smell dysfunction, head and muscle pain, diarrhea) as well as visual and ocular complaints, hematologic and inflammatory laboratory parameters, respiratory rate, and native peripheral oxygen saturation were assessed. Disease severity according to the classification of Wu and McGoogan [21] as well as COVID-19-specific treatment regimens were recorded. Between one and 33 days after COVID-19 symptom onset, a complete and thorough ophthalmic examination was performed including visual acuity, ocular motility, color perception, and intraocular pressure as well as slit-lamp ophthalmoscopy of the anterior and posterior segments. As far as the patients’ conditions allowed it, OCT of the macula and the optic disc was conducted furthermore. A second examination was performed 29 to 192 days later, according to the patient’s availability. Thereby, assessment of systemic symptoms and ophthalmic examination, as described above, were repeated. In some patients, OCT-Angiography (OCT-A) was additionally performed.

Statistical analysis was conducted using Prism 5.0c (GraphPad Software, San Diego, CA, USA). Ophthalmic symptoms and parameters were defined as primary outcomes. Descriptive analysis with calculation of frequencies (%), median (range), and mean (±standard deviation) values was applied. Comparisons were performed using the Student’s t-test for normally distributed data and the Mann–Whitney U test for non-normally distributed data. A *p*-value < 0.05 was deemed significant.

## 3. Results

### 3.1. Patient Characteristics

Of the 50 included patients, 14 were female (28%). Median age at COVID-19 symptom onset was 64.5 (range 29–90) years and median time between symptom onset and hospital admission was 6 (1–33) days. Type 2 diabetes mellitus (T2D) was prevalent in 15 patients (30%), 26 patients (52%) suffered from arterial hypertension (HT), and two patients from immunocompromising diseases (patient #8: renal transplantation; patient #37: diffuse large B-cell lymphoma). SARS-CoV-2 infection was confirmed by PCR in 48 and by serology in two patients. Clinical or radiological signs of pneumonia were evident in 45 patients (90%). COVID-19 disease severity was mild in 15 (30%), severe in 30 (60%), and critical in five cases (10%), with six patients (12%) requiring admission to the intensive care unit (ICU) and three patients (6%) requiring mechanical ventilation. The mortality rate within the observed time period was 6% (*n* = 3 patients). Data is shown in Table S1.

### 3.2. Clinical and Laboratory Parameters at Hospital Admission and COVID-19-Specific Treatment Regimens

Forty-four patients (88%) showed respiratory symptoms such as cough, dyspnea, or sore throat. Twenty-nine patients (58%) exhibited an increased respiratory rate (>20 breaths per minute while resting). In 22 patients (44%), blood oxygen saturation was lower than 94%, and 35 patients (70%) were administered supplemental oxygen. Fever (body temperature ≥38° C) was present in 37 patients (74%). Six patients (12%) reported smell and/or taste dysfunction. Ten patients (20%) complained of headache. Disorientation, nausea, and vertigo were present in individual cases. Data is shown in Table S2.

Median leukocyte count (WBC), lymphocyte count (TLC), and thrombocyte count (PLT) were 5.69 (3.01–17.37) G/L, 1.245 (0.21–34.6) G/L, and 188 (92–617) G/L, respectively, with values being above normal limits in five (10%), ten (20%), and four patients (8%), respectively. In 22 patients (44%), serum hemoglobin (Hb) concentrations were below normal limits (median 134 (84–181) g/L). Procalcitonin (PCT; median 0.08 (0.02–1.9) µg/L) and C-reactive protein (CRP; 73.7 (3–297) mg/L) levels were increased in 29 patients (58%) and 45 patients (90%), respectively. Twenty-two patients (44%) presented with raised serum concentrations of lactate dehydrogenase (LDH; median 310 (52–554) U/L) and 34 patients (68%) with elevated serum levels of alanine aminotransferase (ALAT; median 41 (6.6–635) U/L. Signs of insufficient creatinine clearance were evident in 14 patients (28%). Data is shown in Table S2.

Six patients (12%) were treated with hydroxychloroquine (cumulative dosage of 1400–1600 mg). Three patients (6%) received lopinavir/ritonavir and five patients (10%) remdesivir. In 29 patients (58%), a corticosteroid regimen was established, either isolated or in combination with one of the other mentioned medications. Data is shown in Table S1.

### 3.3. Baseline Ophthalmic Examination

Median time between onset of COVID-19 symptoms and baseline ophthalmic examination was 9 (<1–33) days. Median best corrected visual acuity (BCVA) was 0.1 (0–1.8) Logarithm of the Minimum Angle of Resolution (LogMAR) in the right eye (oculus dexter; OD) as well as in the left eye (oculus sinister; OS) (mean BCVA 0.198 ± 0.32 (±standard deviation) LogMAR OD; mean BCVA 0.19 ± 0.34 LogMAR OS). Median intraocular pressure (IOP) was 16 (8–22) mmHg OD and 16 (11–21) mmHg OS (mean IOP 15.8 ± 3.12 mmHg OD; 16.1 ± 2.74 mmHg OS).

Twenty-one patients (42%) reported a previous ophthalmological history with features ranging from dry eye disease (DED), allergic conjunctivitis, corneal foreign body, ocular trauma and cataract surgery to glaucoma, retinal detachment, diabetic retinopathy (DRP), and retinal vein occlusion (RVO) with subsequent macular edema and intravitreal anti-vascular endothelial growth factor (anti-VEGF) treatment.

Seven patients (14%) reported visual or ocular complaints of recent onset, consisting of photosensitivity, foreign body sensation, and blurred vision (patients #5, #17, #26, #27, #40, #47, #50). Five patients (patients #1, #5, #7, #9, #21) mentioned possibly faded red vision in one eye, one of whom (patient #5) was in combination with ocular discomfort upon eye movement. However, the patients’ symptom descriptions were not consistent with the classical criteria of red desaturation or retrobulbar pain as typically seen in optic neuritis.

In two further patients, increased conjunctival secretion was present (patients #11 and #12), however, no other signs of ocular surface affection such as conjunctival injection, follicles/papillae, or caruncular swelling were notable. Apart from discrete corneal stromal scars in two patients (patients #3 and #16), no corneal abnormalities were detected, in particular, no infiltrates or endothelial precipitates, suggesting keratitis or uveitis. Anterior chambers were screened for cells, pigment, and Tyndall’s phenomenon and the iris for synechiae and transillumination phenomenon. Both anterior chambers and irises presented unremarkable in all eyes. Forty-seven eyes presented relatively clear lenses, while cataract was evident in 43 eyes and 10 eyes were pseudophakic.

In all of the patients, the optic disc, the posterior pole, and the retinal periphery could be accurately inspected by slit-lamp ophthalmoscopy. Thus, a valid assessment of the posterior segment could be achieved in all of the patients. The vitreous body was screened for cells, pigment, and hemorrhages, though was found to be unremarkable in all eyes. In five eyes of three patients (patients #11, #29, and #30), optic discs appeared glaucomatous with an increased cup-to-disc ratio. Apart from the glaucomatous discs in those patients, no signs of optic neuropathy were notable in any of the patients, particularly no optic disc edema, optic disc atrophy, or peripapillary hemorrhages. Except for geographic macular atrophy in a patient with known neovascular wet age-related macular degeneration (wAMD; patient #6), occasional macular drusen, myelinated nerve fibers, and signs of epiretinal membrane (ERM), posterior poles appeared normal in all eyes. In patient #11, who had been previously diagnosed with DRP, multiple retinal hemorrhages and a choroidal nevus were found. In patient #29, who displayed a history of HT, the retinal periphery presented with small hemorrhages in both eyes (oculus uterque; OU). Apart from pigmented laser spots in patient #4 with a history of retinal detachment, no other signs of peripheral retinal abnormalities were detected in any of the patients. Due to the impaired patients’ condition, OCT of the macula and/or the optic disc could be performed in only 27 patients. In one patient with a previous history of HT and RVO, OCT showed correlates of a retinal microaneurysm and macular edema (patient #18). Apart from a small intraretinal cavitation (patient #19), correlates of macular drusen, and ERM, no further macular abnormalities were detected. No signs of optic disc edema or atrophy were found on any image. Complete data on baseline ophthalmic examination is shown in Table S3.

### 3.4. Follow-Up Examination

Twenty-seven patients (54%) completed the follow-up examination. Three patients had deceased (patients #8, #19, #35), one patient was unavailable after suffering a stroke (patient #30); and 19 patients refused to participate. Median time between onset of COVID-19 symptoms and follow-up examination was 55 (38–205) days. Ten patients reported residual respiratory symptoms such as cough, dyspnea, or sore throat. Three patients complained of residual taste or smell dysfunction, while body temperature was normal in all patients. None of the patients reported residual or recently occurred visual or ocular disturbances. Median BCVA was 0.1 (0–1) LogMAR OD and 0 (0–1.8) LogMAR OS (mean BCVA 0.115 ± 0.2 LogMAR OD; mean BCVA 0.13 ± 0.36 LogMAR OS). The same patients’ median BCVA at the first examination was 0.1 (0–0.7) LogMAR OD (*p* = 0.55) and 0.1 (0–1.3) LogMAR OS (*p* = 0.21) (mean BCVA 0.178 ± 0.24 OD; mean BCVA 0.178 ±0.27 LogMAR OS). At follow-up examination, IOP was within normal limits in all eyes. Median IOP was 14 (6–20) mmHg OU (mean IOP 13.32 ± 3 mmHg OD; mean IOP 13.2 ± 2.89 mmHg). At baseline, the same patients’ median IOP was 15 (8–21) mmHg OD and 15 (11–21) mmHg OS (mean IOP 14.84 ± 2.82 mmHg OD (*p* = 0.07); mean IOP 15.36 ± 2.83 mmHg OS (*p* = 0.01)).

Conjunctival follicles/papillae were detected in one patient on both sides (patient #21). Apart from that, no other findings or symptoms suggesting active or previous anterior segment inflammation were present in any of the patients. No signs of cataract formation or progression were evident. In patient #7, posterior segment examination revealed a small peripapillary hemorrhage. In patient #11, who had a previous history of DRP, OCT revealed macular edema. All other findings were consistent with baseline parameters. Complete data on follow-up examination is shown in Tables S4 and S5.

## 4. Discussion

While the presence of SARS-CoV-2 in ocular secretions as well as ocular surface manifestations in COVID-19 are widely recognized and based on relatively solid evidence [3], literature on intraocular involvement is limited and controversial [9,11]. Some reports suggest high incidences of retinal lesions, while other studies did not find any signs of intraocular manifestations [22,23]. Various mechanisms of nervous system and ocular involvement in COVID-19 are under debate including cytokine dysregulation (referred to as “cytokine storm”), vascular endothelial injury and ischemic processes triggered by hypercoagulability, direct viral cytotoxicity as well as secondary damages mediated by aggravation of pre-existing hypoxia and HT [9,11,18,20,24,25,26,27].

Cardiovascular diseases are known to be associated with a number of retinal abnormalities found in patients with COVID-19 such as cotton wool spots or retinal hemorrhages [15]. Pre-existing cardiovascular diseases are also a major risk factor for a severe disease course and poor outcome in COVID-19 [1,28]. As such patients are more prone to develop critical disease requiring hospital admission, and they may also be more likely to be enrolled in studies on intraocular findings.

Pulmonary expression of ACE-2 (the major receptor for SARS-CoV-2), immunocompromised state, obesity, pre-existing endothelial dysfunction, and the tendency to hypercoagulability are all features that render diabetic patients more vulnerable to SARS-CoV-2 [29,30]. Among patients hospitalized for COVID-19, uncontrolled hyperglycemia and new-onset or deterioration of pre-existing T2D frequently occur [28]. Furthermore, corticosteroids, which are recommended for the treatment of patients with COVID-19, are known to trigger hyperglycemia [30]. Moreover, ACE-2 is an inhibitor of the renin–angiotensin–aldosterone system, which is a major mediator in blood pressure regulation. By binding ACE-2 receptors, SARS-CoV-2 potentially drives HT [31].

Our cohort includes hospitalized patients of different age and disease severity groups. Some of them required transfer to the ICU and mechanical ventilation. During the acute phase of the disease, symptoms and signs indicating ocular surface irritation were found in 18% of the patients, which is consistent with previous studies [3]. In contrast, we found very discrete posterior segment lesions presenting as retinal and peripapillary hemorrhages in only three patients. The findings were noted during hospitalization in two patients and at follow-up examination in the other case. Pre-existing cardiovascular diseases were highly prevalent in this cohort. All three mentioned patients had a history of HT or T2D with known end organ damage such as DRP. Retinal hemorrhages are highly unspecific and likely to be associated with these pre-existing diseases. We detected no other signs, suggesting intraocular or optic nerve affection in any of the patients. No significant changes of BCVA were recorded between baseline and follow-up examination and no evidence of cataract formation or progression was found. IOP was within normal limits over the whole observation period and no relevant changes were documented. OCT parameters were widely stable over the whole observation period in all examined subjects.

Lani-Louzada et al. stated that COVID-19-related deterioration of pre-existing systemic conditions most likely caused the retinal lesions found in their patients [18]. We came to the same conclusion regarding our patients. It appears most credible that aggravation of pre-existing hyperglycemia and HT, rather than direct viral cytotoxic effects or inflammatory responses, led to the retinal alterations detected in some of our patients. Our study was limited by the relatively small number of examined patients. Furthermore, the patients were hospitalized at different timepoints relative to the onset of COVID-19 symptoms. A significant proportion of the patients refused to participate in the second ophthalmic examination or, as a result of impaired condition or personal preferences, were only available after very different time periods. Therefore, the patients could not be standardized regarding the time span between disease onset and the ophthalmic examinations. Thus, caution needs to be exercised when interpreting our findings within a general context. Nevertheless, considering the age distribution and the prevalent co-morbidities, our findings represent what would be expected in a random cohort of respective patients without COVID-19, suggesting that routine ophthalmic exams in most patients with COVID-19 is not necessary. This interpretation, however, highlights the importance of close monitoring and prompt treatment of underlying cardiovascular diseases in patients with COVID-19. Furthermore, it is noteworthy that no signs of ocular side effects were found in the six patients who were treated with hydroxychloroquine.

## 5. Conclusions

To the best of our knowledge, this is the largest prospective cohort of patients with COVID-19 to be systematically, comprehensively, and longitudinally screened for intraocular and optic nerve involvement. Direct viral invasion of intraocular tissue and inflammatory responses may be possible in individual cases. However, our findings suggest that SARS-CoV-2 does not regularly affect intraocular tissue and the optic nerve. Hence, closer ophthalmic surveillance and general ophthalmic screening may not be necessary for all patients presenting with COVID-19, regardless of the severity of the disease. Instead, close monitoring and prompt treatment of underlying cardiovascular diseases is warranted.

## Data Availability

The data presented in this study are available in Tables S1–S5.

## Supplementary Materials

The following are available online at https://www.mdpi.com/2077-0383/10/5/896/s1, Table S1: Patient characteristics and medical treatment regimens for COVID-19; Table S2: Symptoms and laboratory findings at hospital admission; Table S3: Ophthalmic findings at hospital admission; Table S4: Ophthalmic findings at follow up examination between 41 and 205 days after COVID-19 symptom onset; Table S5: Systemic symptoms at follow up examination.
